# Empowering episodic memory through a model-based egocentric navigational training

**DOI:** 10.1007/s00426-022-01777-6

**Published:** 2022-12-07

**Authors:** Agustina Fragueiro, Annalisa Tosoni, Rosalia Di Matteo, Giorgia Committeri

**Affiliations:** Department of Neuroscience, Imaging and Clinical Sciences, University “G. D’Annunzio”, ITAB, Institute of Advanced Biomedical Technologies, Via Dei Vestini 33, 66100 Chieti, Italy

**Keywords:** Spatial navigation, Declarative memory, Episodic memory, Path integration, Semantic memory

## Abstract

Recent works have proposed that spatial mechanisms in the hippocampal–entorhinal system might have originally developed to represent distances and positions in the physical space and successively evolved to represent experience and memory in the mental space (Bellmund et al. 2018; Bottini and Doeller 2020). Within this phylogenetic continuity hypothesis (Buzsáki and Moser 2013), mechanisms supporting episodic and semantic memory would have evolved from egocentric and allocentric spatial navigation mechanisms, respectively. Recent studies have described a specific relationship between human performance in egocentric navigation and episodic memory (Committeri et al. 2020; Fragueiro et al. 2021), representing the first behavioral support to this hypothesis. Here, we tested the causal relationship among egocentric navigation and both episodic and semantic components of declarative memory. We conducted two experiments on healthy young adults: in the first experiment, participants were submitted to a navigational training based on path integration, while in the second experiment, participants completed a control training based on visual–perceptual learning. Performance in a set of memory tasks assessing episodic, semantic and short-term memory was compared among the pre- vs. post-training sessions. The results indicated a significant improvement of the episodic memory but not of the semantic or the short-term memory performance following the navigational training. In addition, no modulations of performance across the three memory tasks were observed following the control perceptual training. Our findings provide brand-new evidence of a potential causal association between mechanisms of egocentric navigation and episodic memory, thereby further supporting the phylogenetic continuity hypothesis between navigation and memory mechanisms as well as offering new insights about possible clinical applications of navigational trainings for memory functions/dysfunctions.

**Supplementary Information:**

The online version contains supplementary material available at 10.1007/s00426-022-01777-6.

## Introduction

Converging evidence from neuropsychological studies in amnesic patients and spatial navigation research in rats has traditionally indicated a central role of the medial temporal lobe in both declarative memory and spatial navigation functions (Eichenbaum & Cohen, 2014; Epstein et al., 2017). In accordance with this view, a ground-breaking model of hippocampal functions has been recently formulated supporting the idea of a spatial representational format for high-level cognition (Bellmund et al., 2018). According to this account, spatial codes associated with neural mapping of positions and distance in the physical environment is also assumed to underlie the mapping and organization of conceptual knowledge and memory in the human cognitive system. Within this view, in particular, processing mechanisms in the hippocampal–entorhinal system are assumed to support knowledge and representation of cognitive space spanned by a set of quality dimensions beyond the Euclidean space for navigation.

At a wider evolutionary level, these processing mechanisms in the hippocampal–entorhinal system might have been originally developed to represent distances and positions in the physical space and successively evolved to represent experience and memory in the mental space. Within the phylogenetic continuity hypothesis proposed by Buzsáki and Moser (2013), in particular, high-level mechanisms supporting episodic and semantic memory functions would have respectively evolved from egocentric (i.e., self-based) and allocentric (i.e., map-based) spatial navigation mechanisms (see also Bottini & Doeller, 2020, for a review analysis). Within the same model, moreover, map-based/allocentric navigation would have evolved from self-based/egocentric navigation, thereby making the basic navigational mechanism of homing the core origin of higher-level functions within both the same domain (navigation) ad across domains (from navigation to memory).

Using experimental psychology methods applied to the analysis of human behavior during navigational and memory tasks, we have recently described a statistically specific and predictive relationship between human performance (i.e., accuracy) during egocentric navigation (i.e., path integration performance) and episodic, but not semantic memory tasks (Committeri et al., 2020; Fragueiro et al., 2021). These data represented a first behavioral support to the phylogenetic continuity hypothesis and raised the fascinating possibility that a boosting of the episodic memory abilities could be obtained following a behavioral training in egocentric navigation performance.

Accordingly, the implementation of spatial learning strategies engaging the medial temporal lobe has been shown to represent the basis of superior declarative memory functions (Maguire et al., 2003), but to our knowledge, no study has so far addressed the question by employing a basic training on egocentric navigation to indirectly empower episodic memory performance. Of note, as indicated by spatial navigation research in rodents, the hippocampal formation and the afferent structures have been consistently shown to undergo massive forms of synaptic reorganization during prolonged exposure to complex environments and navigation (e.g., Kempermann et al., 1997; van Praag et al., 2000).

In the present study, inspired by the phylogenetic continuity model (Buzsáki & Moser, 2013) and the supporting experimental evidence on healthy human performance (Committeri et al., 2020; Fragueiro et al., 2021), we examined the hypothesis of beneficial effects of an egocentric navigational training on episodic memory. To this aim, we conducted a first experiment (Experiment 1) on a group of participants undergoing a proprioceptive path integration training collected in-between a memory evaluation session including an episodic memory task based on film-based temporal order memory, a semantic memory task based on semantic categories and a visual short-term memory task (Fragueiro et al., 2021). The pre- and post-training memory sessions were collected using parallel versions of the same memory tasks, allowing pre- vs. post-training comparisons. A second control experiment (Experiment 2) was conducted on a second group of subjects who performed the same versions of the memory tasks before and after a visual–perceptual training. We predicted a specific, causal effect of the navigational training on episodic memory performance but not semantic or short-term memory performance. Furthermore, we expected an improvement of the episodic memory performance following the egocentric navigation but not the perceptual control training.

## Materials and methods

### Memory tasks

Memory performance was evaluated on the episodic, semantic and visual short-term memory tasks employed in Fragueiro et al. (2021). To assess the effect of the training on memory performance, two parallel versions of each task were developed with matching difficulty level. An analysis on the levels of difficulty of the parallel forms of each task can be found in Supplementary Tables 1 and 2, and in Supplementary Fig. 1.

#### 2.1.1. Travel in time task (TT)

**Fig. 1:**
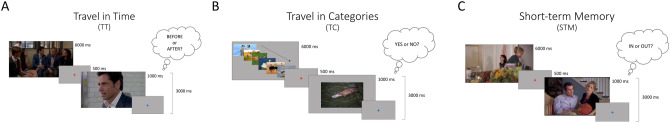
Memory tasks administrated in the pre- and post-training sessions. **A** Travel in Time task (TT) used to assess episodic memory performance; **B** travel in categories task (TC) used to test semantic memory performance; **C** visual Short-Term Memory task (STM)

To assess episodic memory performance, we used a temporal order memory task based on movie scenes. The episodic memory task, hereafter defined as the “Travel in Time” (TT) task included an encoding and a retrieval session separated by a ∼ 40 min interval. Two versions of the TT task were constructed based on two American television series treating the same arguments and inside similar spatial and temporal contexts. At encoding, in particular, a full episode of the series “Grandfathered” (Season 1, Episode 1, “Pilot”; duration: 21:20 min) or “Raising Hope” (Season 1, Episode 1, “Pilot”, duration: 22 min) dubbed in Italian was shown to participants. Both episodes portray character’s real-life-like actions and contain ordinary events (e.g., breakfast or dinner at home, a walk in the park, a visit to the hospital or a supermarket). Participants were instructed to carefully watch the episode but were not informed about the nature of the following tasks. In the retrieval session, participants were instructed to provide temporal order judgments on the encoded audio–visual material. Each trial began with the presentation of a 6 s video clip extracted from the previously encoded episode, followed by a 500 ms red fixation cross and a target picture of 1-s duration (Fig. 1A). Target pictures were extracted from movie scenes occurring 1–2 min earlier or after the onset-offset of the corresponding video clip, with a 1:1 ratio. Participants were provided with 3 s from the onset of the target image to indicate whether it belonged to a scene occurring before or after the video clip (i.e., “z” key for “before”, “m” key for “after”). A 1-s ITI preceded the following trial. The retrieval session included 45 trials and was preceded by 4 practice trials.

#### 2.1.2. Travel in Categories task (TC)

Semantic memory was assessed using a semantic categorization task based on images extracted from the web. Each trial began with the presentation of six consecutive pictures for 6 s (1 s for each picture), followed by a red fixation cross for 500 ms and by a target picture of 1-s duration (Fig. 1B). Participants were provided with 3 s from the target image onset to indicate whether it belonged to the same/different semantic category of the six preceding elements (“m” key for “yes”, “z” key for “no”). A 1-s ITI preceded the following trial. The six elements of the initial stream were selected from a given semantic category, with categories including pictures of both living and nonliving elements (e.g., reptiles, European countries, famous singers, farm animals). Target pictures were either selected from the same or different semantic category of the preceding stream of pictures, with a 1:1 ratio. As an example of a “no” trial, six pictures of flying animals were presented at the beginning of the trial (i.e., a toucan, a hawk, a butterfly, a dragonfly, a bat, an eagle) and a picture of a penguin was presented as target picture. Participants were not explicitly informed about the unifying semantic feature. Instead, they were invited to continuously integrate information during the stream of pictures to update their hypothesis about the category definition (from birds to flying animals in the example). A total of 120 trials were originally developed on a pilot study among which 90 trials of medium–high difficulty were selected for the experiment (45 trials with matched difficulty for each parallel version). Each form included 4 practice and 45 experimental trials.

#### 2.1.3. Short-term memory task (STM)

Given that the two main tasks (TT, TC) were based on online maintenance and updating of visual information from the initial video clip/pictures stream, a visual short-term memory (STM) task was additionally included in the design to control for potential effects of STM on tasks’ performance. Two versions of the STM task were constructed using two American television series treating the same arguments and inside similar spatial and temporal contexts. As for the previously described tasks, the STM task was composed of an initial 6-s video clip extracted from an episode of the television series “Modern Family” (Season 1, Episode 1, “Pilot”; duration: 22 min) or “New Girl” (Season 1, Episode 1, “Pilot”; duration: 23 min), followed by presentation of a red fixation cross for 500 ms and by a target picture of 1-s duration extracted from the same episode (Fig. 1C). Target pictures were extracted from movie scenes included in the presented video clips or from movie scenes occurring 1–2 s earlier or after the onset–offset of the presented video clip, with a 1:1 ratio. Participants were provided with 3 s from the target image onset to indicate whether the target picture was extracted or not from the previously presented video clip (“m” key for “yes”, “z” key for “no”). A 1-s ITI preceded the following trial. Each version of the task included 4 practice trials and 45 experimental trials.

### Experiment 1. Egocentric navigational training

#### Participants

A total of 27 healthy volunteers (mean age = 24.9 $\pm$ 2.6, 16 females), recruited from the University G. d’Annunzio of Chieti-Pescara, participated in the study. All participants were naive as to the purpose of the experiment, reported normal or corrected-to-normal vision, and were enrolled in the study after providing informed consent. None of the participants reported having previously watched the television series “Grandfathered” or “Raising Hope” before the experiment. The study was conducted following the ethical standards of the 1964 Declaration of Helsinki and was approved by the University Ethics Committee (prot. #1932 approved on July 11, 2019).

#### Egocentric navigational training

**Fig. 2:**
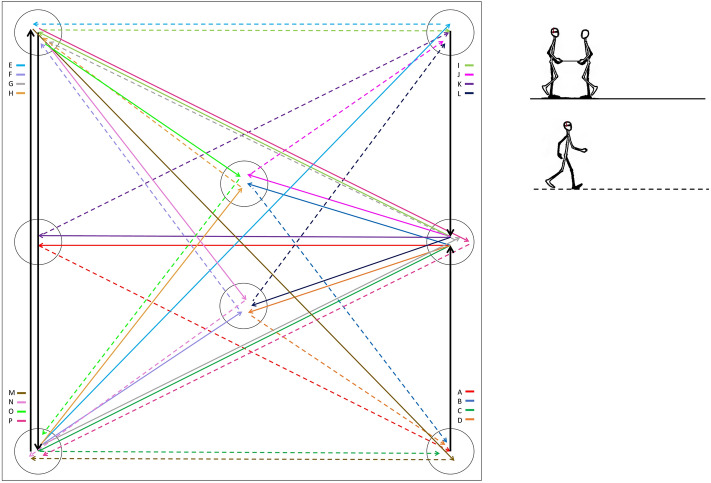
Representation of the carpet (4 × 4mts) with all the paths included in the navigational training. Participants were led by the experimenter along two sides of a triangle (first segment = black continuous lines, second segment = colored continuous lines) before autonomously returning to the homing position (dashed colored lines). *Black arrows* represent the first triangle segment shared by four paths starting in the same circle. The task was adapted from Committeri et al. (2020) and the details of each triangulation are reported in Supplementary Table 3

Spatial navigation abilities were trained on a proprioceptive path integration task adapted from Committeri et al. (2020) and based on the path integration task known as Triangle Completion Task (Wiener et al., 2011). The triangle completion training session was defined by a series of triangulations in which blindfolded participants, wearing headphones emitting white noise, were guided along two sides of a triangle, and then instructed to autonomously return to the starting position, i.e., maintaining in memory the starting position for homing. The experimenter guided the participants along the path holding one end of a stick, while the other end was held by participants with both hands. At the beginning of each trial, the start position was indicated by the experimenter by means of two taps on the participant’s shoulder. The stick was tugged lightly twice to indicate to start walking and tilted upward and rotated to indicate a change of direction. At the end of each path, a slowdown of the bar prompted the participant to orient toward and return to the starting position by following a direct path (Fig. 2).

The carpet (4 × 4 mts) on which the paths were performed contained a total of 16 triangulations differing for: (1) length of the sides of the triangles (first: 175 or 350 cm; second: from 185 to 495 cm), (2) rotation directions (left or right), (3) first turning angle (from 75° to 145°), (4) homing angle (from 90° to 155°), and (5) homing distance (from 210 to 395 cm). The details of each triangulation are reported in Supplementary Table 3.

In each training session, participants completed 6 randomly selected different triangulations, with each triangulation repeated three times consecutively, for a total of 18 trials per session and a total duration of ~ 40 min. After each trial, participants received a visual feedback about the homing error (distance from the correct homing position) and were then once again blindfolded and conducted through random detours to the following starting position.

Performance was measured in each triangulation in terms of distance error from the homing position weighted by the correct homing distance. The criterion for a successful training was set at a maximum of 0.20 error from the mean performance in the third repetitions within each session. The number of sessions required to reach the criterion varied across participants between 1 and 5 (mean = 2.37), and only 2 participants did not reach the criterion. Examples of some participant’s improvement along the training sessions are reported in Supplementary Fig. 2.

#### Protocol

On the first day, participants completed the first session of the 4 memory tasks described above. Specifically, participants were first required to carefully watch an episode of a television series (“Grandfathered” or “Raising Hope”) and, after a 40 min interval, they completed the TT, the TC and the STM tasks in this order. On the second day, participants underwent the navigational training (each session ~ 40 min) which was collected in consecutive, or almost consecutive days, in a dedicated room. As indicated above, the criterion for successful training was determined in each session on the basis of performance (< 0.20 error from the mean performance in the third triangulations), thereby requiring a variable number of sessions for each participant. The post-training session of the memory tasks was administered following the completion of the training program in the same sequential order as in the pre-training memory session. Parallel forms of each task were administrated counterbalanced across participants among the pre- and the post-training sessions. Pre- and post-training sessions were performed on a 17’ LCD computer monitor (1024 × 768 pixels) inside a dark testing room.

#### Statistical analysis

Statistical analyses were conducted on accuracy data in the memory tasks on a total of 25 participants, after excluding 2 participants that did not reach the training criteria within two weeks. Normal distribution of data was evaluated for each task using the Kolmogorov–Smirnov test on IBM SPSS Statistics 25. After confirming the normal distribution of data in the final sample of 25 participants, a 2 × 3 repeated measures ANOVA was conducted with session (pre- vs. post-training) and memory tasks (TT, TC, STM) as factors. The Bonferroni post hoc test was used for statistical comparisons between the mean scores in the different conditions.

### Experiment 2. Control training

#### Participants

A total of 31 healthy volunteers (mean age = 25 $\pm$ 2.08, 19 females) recruited from the University G. d’Annunzio of Chieti-Pescara participated in the study. All participants were naive as to the purpose of the experiment, reported normal or corrected-to-normal vision, and were enrolled in the study after providing informed consent. None of the participants reported having previously watched the television series “Grandfathered” or “Raising Hope” before the experiment. The study was conducted following the ethical standards of the 1964 Declaration of Helsinki and was approved by the University Ethics Committee (prot. #1932 approved on July 11, 2019).

#### Visual–perceptual training

The control training was based on the visual–perceptual learning task described in Baldassarre et al. (2012, 2016). During the training sessions, participants were instructed to attend the left lower visual quadrant and report the presence/absence of a target shape while maintaining central fixation. On each trial, a fixation cross was centrally presented for 200 ms and was followed by presentation of a target (an inverted T) in central vision for 2000 ms and by an array of 12 search stimuli for 150 ms. The array of 12 stimuli was composed of a set of differently oriented Ts stimuli (distracters) with or without the target shape. The target shape appeared randomly in 1 of 3 locations in the left lower visual quadrant, and never in the remaining three quadrants. Subjects were instructed to attend the lower left visual quadrant and indicate the presence/absence of the target shape by pressing the left/right mouse button with the right hand. Each block included 45 trials of which 36 (80%) contained the target and 9 (20%) did not. Participants completed 10 blocks by session (~ 40 min). Performance was calculated in terms of accuracy in each block weighted by the rate of false positive. As described in Baldassarre et al. (2012, 2016), criterion for a successful training was set at a level of performance (i.e., accuracy) higher than 80% (i.e., an error <  = 20%) in 10 consecutive blocks. As for the navigational training, the number of sessions required to achieve the criterion varied across participants between 1 and 6 (mean = 2.48), and 5 participants did not achieve the training criterion. Examples of some participants’ improvement along the training sessions are reported in Supplementary Fig. 3.

#### Protocol

The protocol was the same as for Experiment 1, with the only exception that participants underwent a visual–perceptual rather than a navigational training.

#### Statistical analysis

Statistical analyses were conducted on accuracy data in the memory tasks on a total of 26 participants, after excluding the 5 participants that did not achieve the training criteria. Normal distribution of data was evaluated for each task using the Kolmogorov–Smirnov test on IBM SPSS Statistics 25, and only one outlier was identified from the curve of normal distribution for the STM scores and was excluded from the following analyses. After confirming the normal distribution of the remaining data (*N* = 25), a 2 × 3 repeated-measures ANOVA was conducted with session (pre- vs. post-training) and memory tasks (TT, TC, STM) as factors.

### Comparison between experiments 1 and 2

#### Statistical analysis

To directly compare the results of Experiment 1 and 2, a mixed-model ANOVA was finally conducted on the total sample of 50 participants with training/group (experimental vs. control) as between-subjects factor and session (pre- vs. post-training) and tasks (TT, TC, STM) as within-subjects factors. Planned comparison tests were used to determine the significant differences between the group means in the different conditions.

## Results

### Experiment 1. Egocentric navigational training

The Kolmogorov–Smirnov test indicated that all variables were normally distributed (all tasks D(25) > 0.13, all *p* values > 0.05). As shown by a one-sample t test against the chance level (0.50), an above-chance performance was observed in all tasks (all *p* values < 0.001, descriptive data are reported in Table 1).

**Table 1:** Mean accuracies and standard deviations of memory tasks in the Navigational Training group

	Pre-training session			Post-training session		
	TT	TC	STM	TT	TC	STM
Mean accuracy	0.67	0.77	0.78	0.76	0.80	0.78
Std. deviation	0.11	0.08	0.04	0.08	0.07	0.05

*TT* travel in time, *TC* travel in categories, *STM* short-term memory

**Fig. 3:**
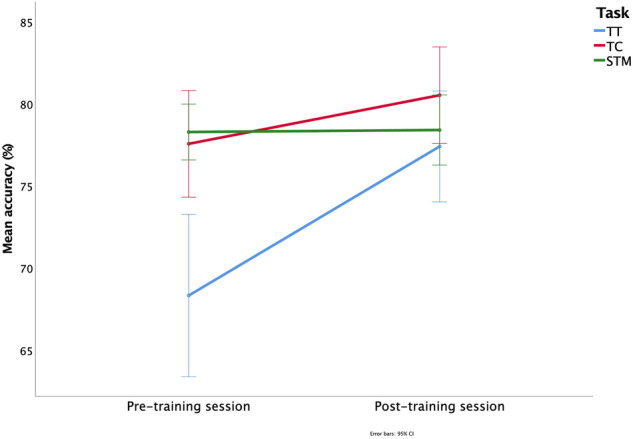
Navigational Training (Experiment 1). Repeated-measures ANOVA including session (pre- vs. post-training) and memory task (TT, TC, STM) as independent factors, and accuracy (%) as dependent variable. A significant difference between session 1 and 2 was only observed for the TT (Travel in Time task, *p* < 0.001)

As illustrated in Fig. 3, the ANOVA results indicated a main effect of session (*F*_(1, 24)_ = 20.01, *p* < 0.001), a main effect of task (*F*_(2, 48)_ = 10.15, *p* < 0.001) and a significant session x task interaction (*F*_(2, 48)_ = 5.49, *p* = 0.007). Consistent with the predictions outlined in the Introduction, the Bonferroni post hoc test confirmed that the effect of the navigational training (pre- vs. post-training session) was statistically significant for the TT (*p* < 0.001) but not for the TC and the STM task (both TC and STM: *p* = 1).

### Experiment 2. Control training

The Kolmogorov–Smirnov test indicated that all variables were normally distributed (all tasks D(25) > 0.11, all *p* values > 0.05). Performance was above chance in all tasks (descriptive data in Table 2), as confirmed by a one-sample t test against chance performance set at 0.50 (all *p* values < 0.001).

**Table 2:** Mean accuracies and standard deviations of memory tasks in the Control Training group

	Pre-training session			Post-training session		
	TT	TC	STM	TT	TC	STM
Mean	0.67	0.75	0.77	0.71	0.79	0.77
Std. deviation	0.11	0.10	0.06	0.17	0.08	0.08

*TT* travel in time, *TC* travel in categories, *STM* short-term memory

**Fig. 4:**
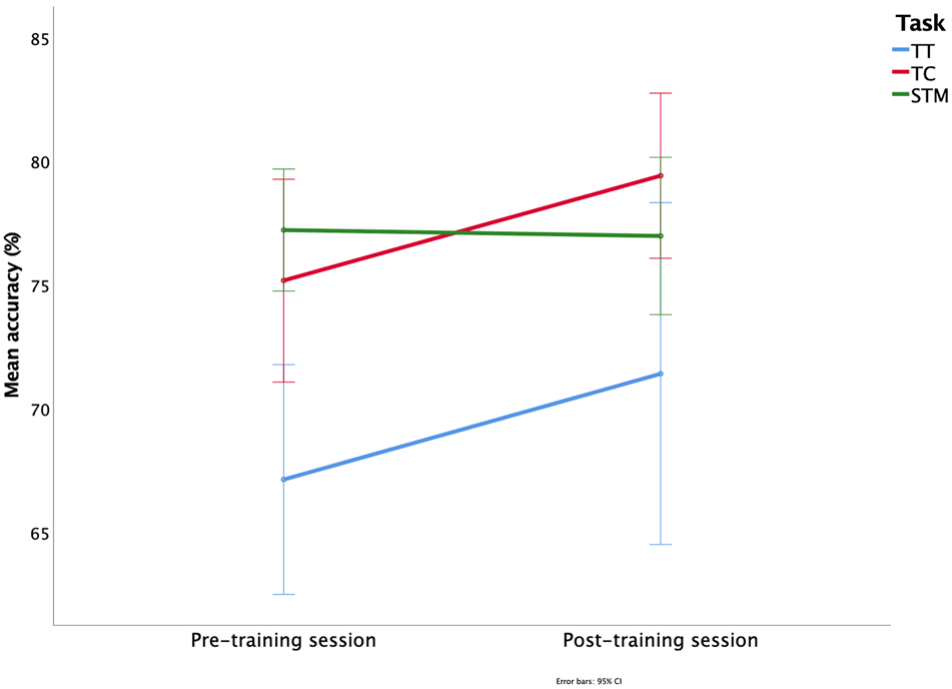
Control Training (Experiment 2). Repeated-measures ANOVA including session (pre- vs. post-training) and memory task (TT, TC, STM) as independent factors, and accuracy (%) as dependent variable. No significant differences were observed between pre- and post-training sessions for any task

As illustrated in Fig. 4, the ANOVA indicated a main effect of task (*F*_(2, 48)_ = 8.823, *p* < 0.001), but a no-significant main effect of session (*F*_(1, 24)_ = 3.436, *p* = 0.08) nor a task x session interaction (*F*_(2, 48)_ = 1.062, *p* = 0.35).

### Comparison between trainings

As illustrated in Supplementary Fig. 4, the results of the mixed-model analysis directly comparing the two training sessions/groups indicated a main effect of session (*F*_(1,48)_ = 15.279, *p* < 0.001), a main effect of task (*F*_(2,96)_ = 18.071, *p* < 0.001) as well as a statistically significant session × task interaction (*F*_(2,96)_ = 4.477, *p* = 0.014), but a no-significant three-way-interaction effect (training/group × session × task) (*F*_(2,96)_ = 0.973, *p* = 0.38). However, based on the results of Experiment 1 and 2 as well as our a priori hypothesis about a specific effect of the navigational training on the episodic memory performance, a series of planned comparison tests were conducted in which the pre- vs. post- sessions of the navigational and the perceptual training were compared across the three tasks. In accordance with our hypothesis, a significant modulation of performance was observed for the episodic memory task following the navigational training but not following the perceptual training, and no significant modulations were observed for the semantic and working memory tasks following both types of training (pre- vs. post-navigational training, TT: *F* = 13.3, *p* = 0.0006; SM: *F* = 1.48; *p* = 0.23; WM: *F* = 0.005, *p* = 0.94; pre- vs. post-perceptual training, TT: *F* = 2.95, *p* = 0.092; SM: *F* = 3.03; *p* = 0.088; WM: *F* = 0.02, *p* = 0.88).

## Discussion

Based on our previous findings of a specific and predictive relationship between self-based human abilities in path integration and temporal order memory tasks (Committeri et al., 2020; Fragueiro et al., 2021), here we tested whether a causal relationship could be described between human processing of egocentric spatial navigation and episodic memory. This question was originally raised from a pivotal work by Buzsáki and Moser on the neurophysiologically derived model of a phylogenetic continuity between mechanisms of navigation in the physical and mental space (memory) (Buzsáki & Moser, 2013). According to this model, in particular, the organizational principles underlying declarative memory would have inherited the fundamental distinction between the spatial reference systems (self-based vs. map-based), supporting navigation in the physical world. Within this view, therefore, episodic memory functions would have evolved from egocentric/self-based navigation and semantic memory functions from allocentric/map-based navigation.

In accordance with this model, and in particular with the egocentric navigation/episodic memory edge of the model predictions, the present results indicated a significant improvement of the episodic memory but not of the semantic or short-term memory performance following an egocentric navigational training. In contrast, no modulations of performance were observed in any of the three memory tasks following a perceptual training on visual discrimination of target shapes. These results suggest that the observed empowerment of the episodic memory performance following the navigational training was associated with the specific properties of the egocentric training and not solely explained by unspecific modulatory effects of task repetition and/or cognitive training. These findings, therefore, provide a general support to the phylogenetic continuity hypothesis between mechanisms of spatial navigation and memory.

In humans, few recent studies have reported improvements in declarative memory performance following a navigational training. A single case study on a patient suffering from topographical disorientation, for example, has described a secondary positive effect of an imagery-based navigational training on episodic memory (Boccia et al., 2019). At the group level, instead, an improvement of long-term memory capabilities (recognition memory) has been observed in older individuals following a training program on a wayfinding game in virtual reality (Wais et al, 2021). In this study, an immersive, complex environment was employed, in which, as for navigational rodent model studies, participants were requested to navigate in novel and unfamiliar surroundings to complete assigned errands and wayfinding. In this case, advancement in performance was probably based on efficient transformation of navigational strategies and environmental representations from egocentric (route-based) to allocentric (survey-based).

Differently from these studies, here we showed a specific modulation of the episodic memory performance following an egocentric navigational training and we speculate that the observed effect might be explained by a core processing similarity between the episodic memory task based on temporal order memory for complex audio–visual material (i.e., movies) and the path integration task based on a continuous spatial updating of both angular displacement and distance from a reference point within an environment (Loomis et al., 1999).

Accordingly, Buzsáki and Moser (2013) have proposed that the mechanisms for representing a path through an environment are basically the same for representing episodes in memory, and the capacity of the brain to generate and store sequences seems to be the key mechanism supporting both self-based navigation and episodic memory. More specifically, as the position-dependent sequential firing of neurons along a linear path, sequences linking arbitrary items in episodic memory are essentially unidimensional. At the neurophysiological level, this shared mechanism for generating neural sequences would be supported by theta phase-modulation of gamma power in the hippocampus and entorhinal cortex (Buzsáki and Moser, 2013; Colgin et al., 2009). Within this view, therefore, the continuous spatial updating of the self-position over time during path integration might represent the basic spatial code for the temporal processing of episodes/events. In this respect, it is worth noticing that the proprioceptive path integration training employed in our study was based on a continuous updating of the self-position from proprioceptive/idiothetic and vestibular information, which might be assumed to reinforce the spatial awareness of the body movement in space, and then provide a refined self-based reference system during subsequent temporal order memory judgments. As far as the specific type of navigational training, finally, we might not exclude that also other forms of egocentric navigational training, such as couplings between a recognition point and the direction in which the route continues, might induce a modulation of the episodic memory performance but we argue that the sequential updating aspect of the path integration training might represent the core mechanism supporting the observed modulation on the memory performance.

The findings presented in the current study suggest a possible booster effect of proprioceptive path integration training on temporal order memory for episodic details. These findings not only further support the model of a phylogenetic continuity between egocentric navigation and episodic memory functions but also provide new insights for possible clinical applications of the egocentric navigational training in the field of memory deficits. We suggest, in particular, that individuals with episodic memory deficits might benefit from an egocentric navigational training for the empowerment and possibly recovery of episodic memory abilities. In line with this hypothesis, deficits in self-based navigation abilities, also specifically involving the path integration performance, have been reported in healthy aging (Allen et al., 2004; Skolimowska et al., 2011; Xie et al., 2017), mild cognitive impairment and Alzheimer’s disease (Mokrisova et al., 2016). Deficits in path integration abilities have been previously associated with compromised entorhinal grid cell computations (Stangl et al., 2018), and both path integration and grid cells deficits have been proposed as sensitive biomarkers of pathological decline in early Alzheimer’s disease (Bierbrauer et al., 2020; Howett et al., 2019; Kunz et al., 2015; Segen et al., 2022). Egocentric heading based on path integration, moreover, has also been thought to more closely support the clinical discrimination between Alzheimer’s and non-Alzheimer’s dementia than navigational tasks based on allocentric, map-based knowledge (Tu et al., 2017). Future research might test the potential effects of a self-based navigational training on older adults and patients with memory deficits. Within this context, it is worth mentioning that, compared to young adults, task performance in healthy elderly people has been thought to more heavily rely on visual processing components rather than on bodily (i.e., tactile, kinematic, proprioceptive) factors, leading to the conclusion that older adults are less embodied than young adults (Costello & Bloesch, 2017). Within this framework, we speculate that a proprioceptive path integration training might also provide a clinical tool for minimizing age-related deficits in multimodal integration and embodied cognition in addition to memory functions.

As far as possible limitations of our study, we acknowledge that the episodic memory task was slightly more difficult than the semantic and short-term memory tasks. However, all three memory tasks were specifically developed with a medium–high difficulty level to avoid roof effects and, therefore, training-induced modulations of performance could be potentially observed for any of the three tasks. Accordingly, a modulation of performance in the post- vs. the pre-training session was also observed for the semantic memory task, but the analyses indicated no statistical significance of the effect. Moreover, the lack of a statistically significant three-way interaction in the mixed-model ANOVA imposes caution on conclusions about a strong causal-specific effect of the navigational vs. the perceptual training. On this basis, therefore, we acknowledge that our results can be more properly thought of as an initial and preliminary, rather than a definitive evidence of a beneficial effect of an egocentric navigational training on episodic memory performance, which could be exploited for potential applications in the clinical field. At a more theoretical level, finally, we acknowledge that our study specifically focuses on the egocentric navigation/episodic memory edge of the phylogenetic continuity model proposed by Buszáki and Moser (2013) and no definitive knowledge has been acquired so far about the association and causal relationship between allocentric/map-based navigation and semantic memory functions. Future studies, therefore, will hopefully conduct specific examinations on this point as well as on possible higher-order interactions between the 4 model components.

In conclusion, the present data offer the first causal evidence for the hypothesis of a phylogenetic continuity between spatial navigation and declarative memory, showing that navigation through time in mental space shares core processing mechanisms with egocentric navigation in the physical space. At the same time, the data offer a new perspective toward clinical applications because training the core, egocentric/self-based navigation through path integration could potentially have boosting effects on episodic, temporally-based memory in both physiological and pathological aging.

## Supplementary Information

Below is the link to the electronic supplementary material.

Supplementary file1 (DOCX 366 KB)

## Data Availability

The datasets generated during the current study are available from the corresponding author on reasonable request.
