# Supplementary material for: Empowering episodic memory through a model-based egocentric navigational training
Source: Psychol Res. 2022 Dec 7;87(6):1743–52. doi: 10.1007/s00426-022-01777-6 (PMC10366265; doi:10.1007/s00426-022-01777-6)
Supplement: Supplementary file 1 — Supplementary file1 (DOCX 366 KB) [file 426_2022_1777_MOESM1_ESM.docx]

**Supplementary Materials**

| Task | Form | Mean | Std. Deviation | Std. Error Mean |
| --- | --- | --- | --- | --- |
| TT | A | 0.71 | 0.14 | 0.02 |
|  | B | 0.71 | 0.12 | 0.02 |
| TC | A | 0.78 | 0.09 | 0.01 |
|  | B | 0.79 | 0.08 | 0.01 |
| STM | A | 0.78 | 0.05 | 0.01 |
|  | B | 0.77 | 0.07 | 0.01 |

**Supplementary Table 1.** Descriptive analysis of the parallel versions of the three memory tasks (N=50).

| Task | t | Sig. (2-tailed) | Mean Difference |
| --- | --- | --- | --- |
| TT | 0.263 | 0.79 | 0.01 |
| TC | -0.733 | 0.46 | -0.01 |
| STM | 0.597 | 0.55 | 0.01 |

**Supplementary Table 2.** Independent samples t-tests confronting both versions of each memory task (N=50).

| **TRIAL** | **1st segment** | **1st turn angle** | **2nd segment** | **Return turn angle** | **Homing distance** |
| --- | --- | --- | --- | --- | --- |
| A | 175 | -90 | 350 | -155 | 395 |
| B | 175 | -75 | 185 | -135 | 285 |
| C | 175 | -125 | 395 | -140 | 350 |
| D | 175 | -100 | 185 | -120 | 210 |
| E | 350 | -135 | 495 | -125 | 350 |
| F | 350 | -120 | 215 | -90 | 290 |
| G | 350 | -110 | 395 | -110 | 395 |
| H | 350 | -145 | 290 | -90 | 215 |
| I | 175 | 125 | 395 | 140 | 350 |
| J | 175 | 100 | 185 | 120 | 210 |
| K | 175 | 90 | 350 | 155 | 395 |
| L | 175 | 75 | 185 | 135 | 285 |
| M | 350 | 135 | 495 | 125 | 350 |
| N | 350 | 145 | 290 | 90 | 215 |
| O | 350 | 120 | 215 | 90 | 290 |
| P | 350 | 110 | 395 | 110 | 395 |

**Supplementary Table 3.** Measures of the paths included in the egocentric navigational training. Distance is reported in centimeters and angles in degrees.


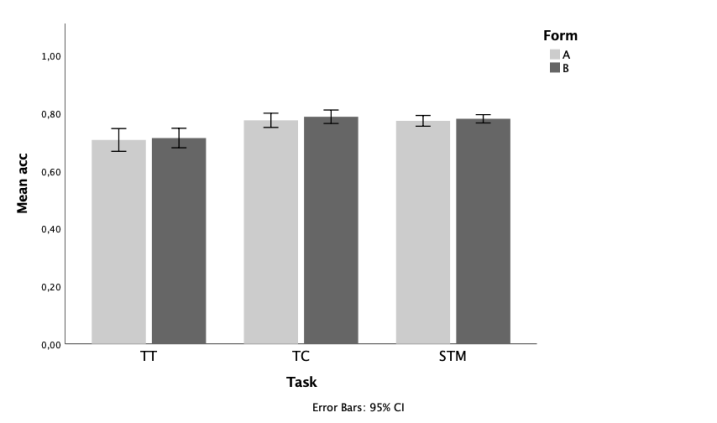


**Supplementary Figure 1.** Mean accuracy for each parallel version of the three memory tasks (N=50).


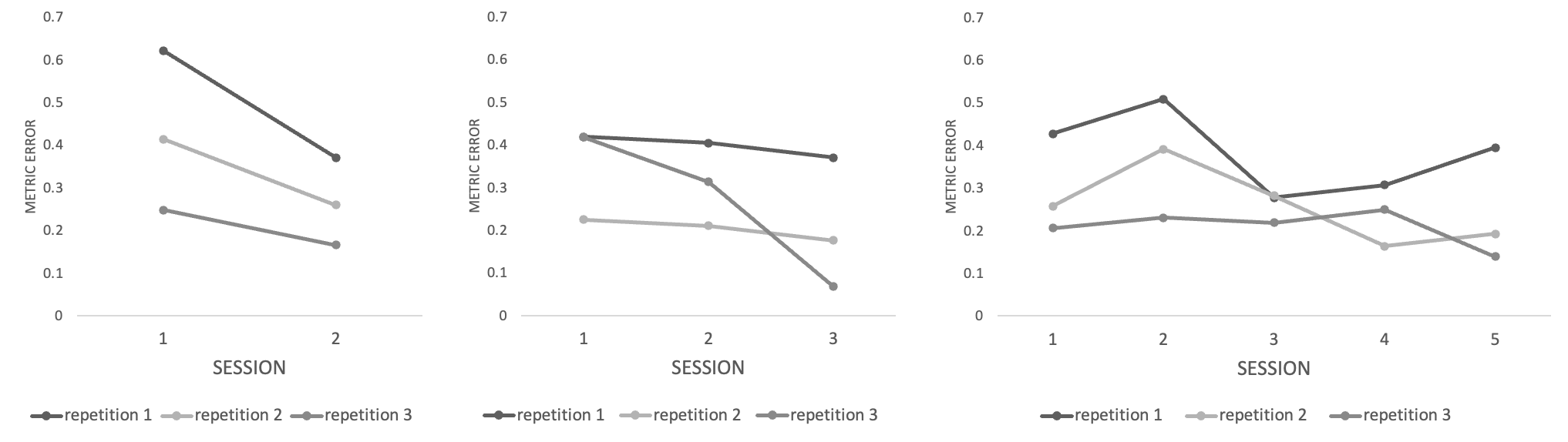


**Supplementary Figure 2.** Examples of behavioral progression during the navigational training in three exemplar participants. Plotted values represent mean distance errors for each repetition (N=3) within a session.


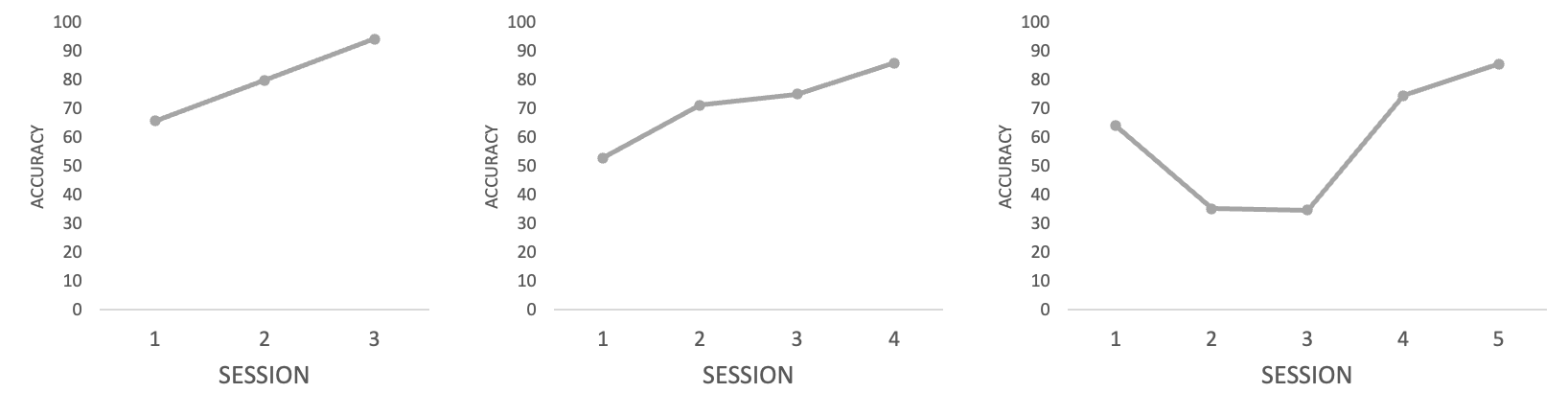


**Supplementary Figure 3.** Examples of behavioral progression during the visual perceptual learning in three exemplar participants. Plotted values represent mean accuracy during the different training sessions.


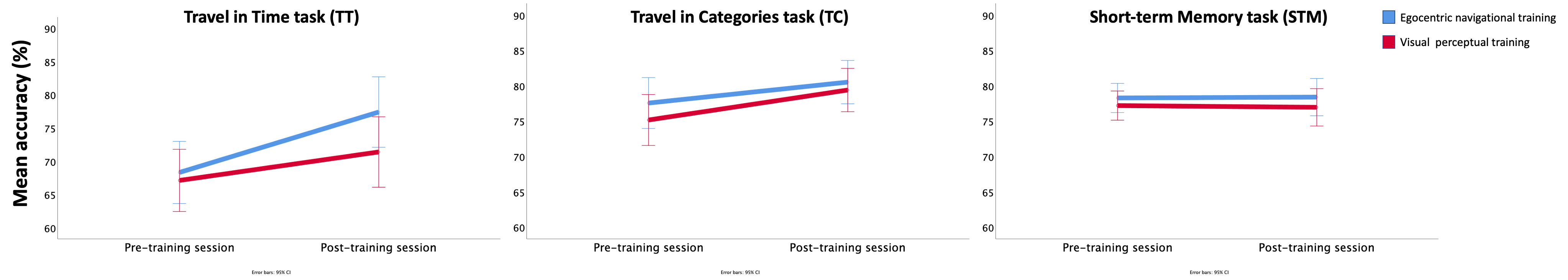


**Supplementary Figure 4.** Mixed model comparing Experiments 1 and 2, including the group (experimental vs. control) as a between factor, session and task as within factors, and accuracy (%) as dependent variable.
